# Methoxyflavones from Black Ginger (*Kaempferia parviflora* Wall. ex Baker) and their Inhibitory Effect on Melanogenesis in B16F10 Mouse Melanoma Cells

**DOI:** 10.3390/plants12051183

**Published:** 2023-03-05

**Authors:** Chen Huo, Sullim Lee, Min Jeong Yoo, Bum Soo Lee, Yoon Seo Jang, Ho Kyong Kim, Seulah Lee, Han Yong Bae, Ki Hyun Kim

**Affiliations:** 1 School of Pharmacy, Sungkyunkwan University, Suwon 16419, Republic of Korea; 2 Department of Life Science, College of Bio-Nano Technology, Gachon University, Seongnam 13120, Republic of Korea; 3 STL Company, Yongin 17086, Republic of Korea; 4 Department of Oriental Medicine Biotechnology, College of Life Sciences, Kyung Hee University, Yongin 17104, Republic of Korea; 5 Department of Chemistry, Sungkyunkwan University, Suwon 16419, Republic of Korea

**Keywords:** *Kaempferia parviflora*, Zingiberaceae, Methoxyflavones, Anti-melanogenesis, B16F10 mouse melanoma cells, SAR

## Abstract

*Kaempferia parviflora* Wall. ex Baker (Zingiberaceae), commonly known as Thai ginseng or black ginger, is a tropical medicinal plant in many regions. It has been traditionally used to treat various ailments, including ulcers, dysentery, gout, allergies, abscesses, and osteoarthritis. As part of our ongoing phytochemical study aimed at discovering bioactive natural products, we investigated potential bioactive methoxyflavones from *K. parviflora* rhizomes. Phytochemical analysis aided by liquid chromatography–mass spectrometry (LC-MS) led to the isolation of six methoxyflavones (**1**–**6**) from the *n*-hexane fraction of the methanolic extract of *K. parviflora* rhizomes. The isolated compounds were structurally determined to be 3,7-dimethoxy-5-hydroxyflavone (**1**), 5-hydroxy-7-methoxyflavone (**2**), 7,4′-dimethylapigenin (**3**), 3,5,7-trimethoxyflavone (**4**), 3,7,4′-trimethylkaempferol (**5**), and 5-hydroxy-3,7,3′,4′-tetramethoxyflavone (**6**), based on NMR data and LC-MS analysis. All of the isolated compounds were evaluated for their anti-melanogenic activities. In the activity assay, 7,4′-dimethylapigenin (**3**) and 3,5,7-trimethoxyflavone (**4**) significantly inhibited tyrosinase activity and melanin content in IBMX-stimulated B16F10 cells. In addition, structure–activity relationship analysis revealed that the methoxy group at C-5 in methoxyflavones is key to their anti-melanogenic activity. This study experimentally demonstrated that *K. parviflora* rhizomes are rich in methoxyflavones and can be a valuable natural resource for anti-melanogenic compounds.

## 1. Introduction

The amount and distribution of melanin, a pigment present in the skin epidermis, are decisive factors in determining skin color. Melanin plays an important role in protecting the skin from ultraviolet rays and harmful external factors [1,2,3]. However, the excessive production and accumulation of melanin in the skin causes spots and freckles. In addition, melanin precursors can cause cell death due to toxicity and diseases, such as skin cancer [4]. The enzymes involved in melanin synthesis include tyrosinase, tyrosinase-related protein-1 (TRP-1), and dopachrome tautomerase (TRP-2). Tyrosinase acts in the initial reaction, the rate-determining step of melanin synthesis, and oxidizes tyrosine to DOPA-quinone via 3,4-dihydroxyphenylalanine (DOPA) [5,6,7]. DOPA-quinone is converted to dopachrome without a catalytic reaction and is converted to 5,6-dihydroxyindole-2-carboxylic acid (DHICA) by the catalyst TRP-2. DHICA is converted to indole-5,6-quinone-2-carboxylic acid by the catalyst TRP-1, which converts it to melanin [5,7,8]. Therefore, the inhibition of tyrosinase, TRP-1, and TRP-2, which catalyze melanogenesis, is an important target for anti-melanogenic activities.

Phenol derivatives, such as hydroquinone, resorcinol, l-ascorbic acid and its derivatives, arbutin, lactic acid, glucosamine, and tunicamycin, have been developed as representative melanin production inhibitors; however, their use is strictly limited owing to problems such as skin irritation and safety concerns [9,10,11,12,13,14]. Therefore, research is being actively conducted to identify safe and effective natural whitening agents.

*Kaempferia parviflora* Wall. ex Baker, known as Thai ginseng or black ginger, belongs to the family Zingiberaceae and is widely distributed in northern Thailand [15]. According to past efficacy and safety evaluations, traditional medicines derived from the rhizome of *K. parviflora* can be used to treat hypertension, inflammation, peptic and colic disorders, allergy, osteoarthritis, and duodenal ulcers [16,17,18]. In addition, *K. parviflora* extract has a wide range of pharmacological effects, including antioxidant, anti-inflammatory, antitumor, cardioprotective, antiallergic, and antibacterial activities [19]. Phytochemical investigations of *K. parviflora* have led to the identification of several biologically active compounds, such as isopimarane, labdane- and clerodane-type diterpenoids, phenolic acids, flavonoids, and steroids [20]. Moreover, *K. parviflora* rhizome extracts have been highlighted to contain flavonoids that exhibit potent biological activities, including antioxidant, neuroprotective, and cognition-enhancing effects [21].

The major components of *K. parviflora* rhizomes are methoxyflavones, structurally identified as 5,7-dimethoxyflavone, 5,7,4′-trimethoxyflavone, and 3,5,7,3′,4′-pentamethoxyflavone [18,22,23], the pharmacokinetic characteristics of which have been investigated [20,24]. In a previous study, 5,7-dimethoxyflavone was shown to reduce the viability of HepG2 cancer cells with an IC_50_ of 25 μM by generating reactive oxygen species and significantly reducing the mitochondrial membrane potential, suggesting that it might be considered to be an anti-liver cancer lead compound [25]. In another study, 5,7,4′-trimethoxyflavone exhibited anti-plasmodial activity against *Plasmodium falciparum*, indicating the possibilities of development as a treatment agent for the malaria parasite [21]. According to a recent study, 3,5,7,3′,4′-pentamethoxyflavone had a relaxing effect on isolated human corpus cavernosum tissue during a sex change operation [26], indicating the potential of this compound as an effective agent to stimulate sexual activity in men. Another methoxyflavone isolated from this plant, 5-hydroxy-3,7,3′,4′-tetramethoxyflavone, was examined for its inhibitory activity against nitric oxide production and exhibited potent anti-inflammatory activity [27]. Considering the biological activities of these methoxyflavones from *K. parviflora* rhizomes, it is essential to investigate methoxyflavone derivatives from this plant to develop novel therapeutics.

As part of continuing natural product discovery research for new bioactive constituents from interesting natural resources [28,29,30,31,32], we investigated potential bioactive flavonoids from *K. parviflora* rhizomes. In our recent study on *K. parviflora* rhizomes, we found that methoxyflavones inhibit tumor necrosis factor-α-induced interstitial collagenase (MMP-1) in human dermal fibroblasts. Among them, 3,5,7-trimethoxyflavone inhibits the pro-inflammatory cytokines interleukin (IL)-1β, IL-6, and IL-8, thus counteracting skin damage [33]. As part of an ongoing study on the discovery of bioactive phytochemicals with beneficial cosmetic properties from *K. parviflora* rhizomes, we isolated six methoxyflavones (**1**–**6**) from the methanolic extract of these rhizomes using column chromatography and high-performance liquid chromatography (HPLC) purification coupled with liquid chromatography–mass spectrometry (LC-MS) analysis. The isolated compounds were tested for their anti-melanogenic activity in B16F10 mouse melanoma cells, and their structure–activity relationships (SARs) were investigated. Herein, we describe the separation and structural elucidation of Compounds **1**–**6**, the evaluation of their anti-melanogenic activity, and SARs. 

## 2. Results and Discussion

### 2.1. Isolation and Structural Identification of Compounds

**Figure 1:**
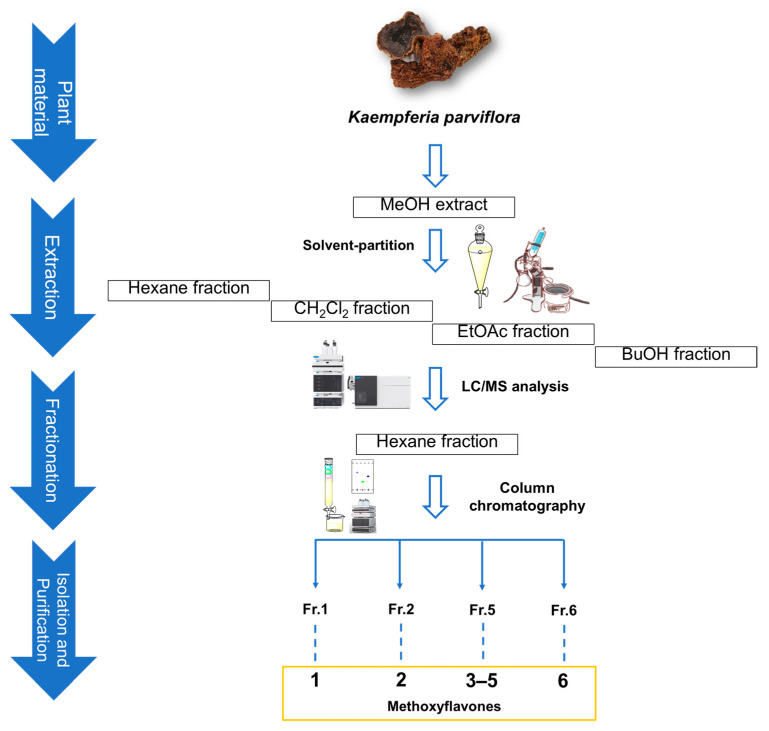
Separation scheme of Compounds **1**–**6**.

**Figure 2:**
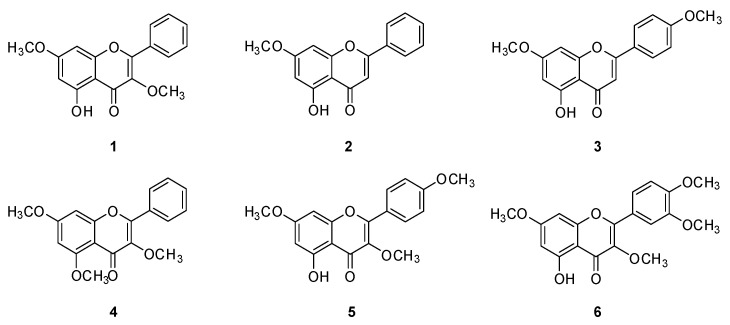
Chemical structures of Compounds **1**–**6**.

The extraction of the rhizomes of *K. parviflora* with 80% MeOH to give the resultant MeOH extract, and then the MeOH extract, was effectively partitioned with four different organic solvents to obtain four main fractions. Each fraction was evaporated to dryness in vacuo to give the following yields: hexane (1.0 g), dichloromethane (CH_2_Cl_2_, 3.2 g), ethyl acetate (EtOAc, 0.4 g), and *n*-butanol (BuOH, 0.5 g)-soluble fractions (Figure 1). Each fraction was analyzed using a house-built UV library database in our LC-MS system, which verified that the hexane fraction was rich in flavonoids. Column chromatography and semi-preparative HPLC separation were efficiently applied, leading to the isolation of six structurally related methoxyflavones (Figure 1). These methoxyflavones were determined to be 3,7-dimethoxy-5-hydroxyflavone (**1**) [34], 5-hydroxy-7-methoxyflavone (**2**) [35], 7,4′-dimethylapigenin (**3**) [36], 3,5,7-trimethoxyflavone (**4**) [37], 3,7,4′-trimethylkaempferol (**5**) [22], and 5-hydroxy-3,7,3′,4′-tetramethoxyflavone (**6**) [38] (Figure 2) by comparing their 1D nuclear magnetic resonance (NMR) spectroscopic data (Figures S1, S3, S5, S7, S9 and S11) with those previously reported and MS data obtained from LC-MS analyses (Figures S2, S4, S6, S8, S10 and S12).

### 2.2. Effects of Methoxyflavones ***1***–***6*** on Viability of B16F10 Mouse Melanoma Cells

**Figure 3:**
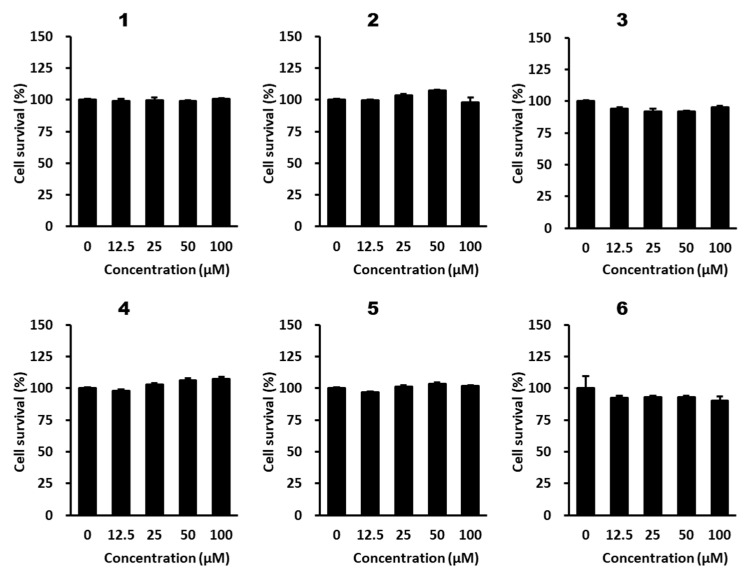
Effects of methoxyflavones **1**–**6** on B16F10 cell viability. B16F10 cells were seeded in 96-well cell culture plates with clear bottoms (5 × 10^3^ cells/well) and were incubated for 24 h. The cells were then treated with the indicated concentrations of methoxyflavones **1**–**6** for 24 h. Cell viability was evaluated using an EZ-Cytox kit. The results are presented as mean ± SEM (*n* =3).

We evaluated the inhibitory effects of the isolated methoxyflavones **1**–**6** on 3-isobutyl-1-methylxanthine (IBMX)-induced melanogenesis in B16F10 cells. Before the evaluation, the effect of each methoxyflavone on B16F10 cell viability was examined. B16F10 cells were treated with methoxyflavones at 12.5, 25, 50, and 100 μM for 24 h. No differences in cell viability were observed between the methoxyflavone-treated and control groups (Figure 3). Therefore, the concentration range of 25–100 μM was selected for further experiments.

### 2.3. Inhibitory Effect of Methoxyflavones ***1***–***6*** on Melanogenesis in B16F10 Mouse Melanoma Cells

Melanin increases the l-tyrosine to l-DOPA ratio by activating tyrosinase in melanocytes and synthesizing l-DOPA-quinone, TRP-2, and TRP-1, which are finally transformed into red-type eumelanin or brown-type pheomelanin [39,40]. Melanin hyperproduction is caused by the increased oxidative stress induced by external stimuli. 

Oxidative stress oxidizes DNA and proteins and causes lipid peroxidation, which plays a major role in increasing the proportion of unsaturated fatty acids. In addition, these stresses excessively increase melanin synthesis and pigmentation in skin melanocytes and contribute to the development of skin cancer [41,42]. Similarly to these oxidative stresses, IBMX inhibits phosphodiesterase, increases cAMP levels, and activates the ERK and PI3K/Akt signaling pathways. These changes promote the production of melanogenesis-related proteins and induce melanin hyperproduction [43].

**Figure 4:**
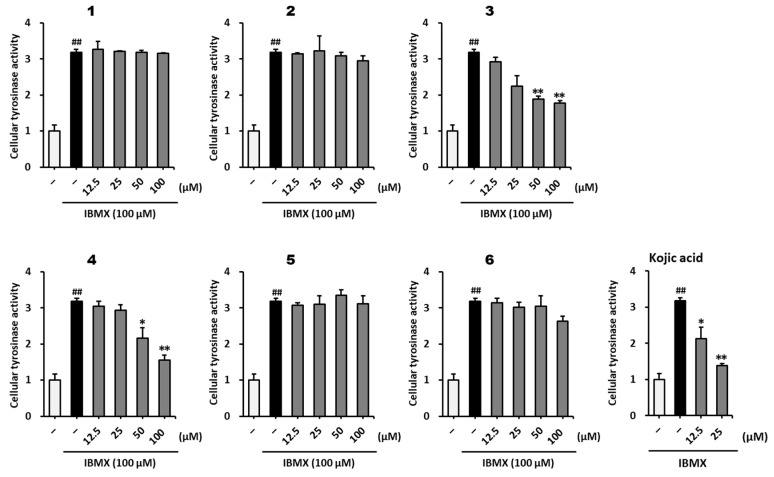
Effect of methoxyflavones **1**–**6** on cellular tyrosinase activity in B16F10 cells. B16F10 cells were seeded in a 60 mm dish at a density of 5 × 10^5^ cells and incubated for 24 h. The cells were then incubated with IBMX and the indicated concentrations of methoxyflavones **1**–**6** for 72 h. Cellular tyrosinase activity was evaluated using l-3,4-dihydroxyphenylalanine (l-DOPA). Kojic acid was used as a positive control. The results are presented as the mean ± SEM (*n* =3). ^##^ *p* < 0.01 compared with the untreated group. * *p* < 0.05 and ** *p* < 0.01 compared to the IBMX-treated group.

The anti-melanogenic effects of methoxyflavones **1**–**6** on IBMX-induced melanogenesis in B16F10 melanoma cells were investigated. As shown in Figure 4, methoxyflavones **3** and **4** decreased cellular tyrosinase activity in IBMX-stimulated B16F10 cells. The IBMX-stimulated group showed a 3.18 ± 0.06-fold (*p* < 0.01) increase in tyrosinase activity compared to that in the vehicle group. Tyrosinase activity decreased in the positive control group treated with kojic acid at 12.5 μM (2.13 ± 0.31-fold, *p* < 0.05) and 25 μM (1.38 ± 0.06-fold, *p* < 0.01) compared with that in the IBMX-treated group. Compound **3** significantly decreased the tyrosinase activity at 25–100 μM (25 μM: 2.25 ± 0.29-fold; 50 μM: 1.88 ± 0.08-fold, *p* < 0.01; 100 μM: 1.78 ± 0.07-fold, *p* < 0.01) compared to that in the IBMX-stimulated group. In addition, Compound **4** decreased tyrosinase activity at 50 μM (2.16 ± 0.29-fold, *p* < 0.05) and 100 μM (1.56 ± 0.13-fold, *p* < 0.01) compared to that in the IBMX-stimulated group. These results indicate that 7,4′-dimethylapigenin (**3**) and 3,5,7-trimethoxyflavone (**4**) significantly inhibited the IBMX-stimulated hyperactivity of tyrosinase. 

**Figure 5:**
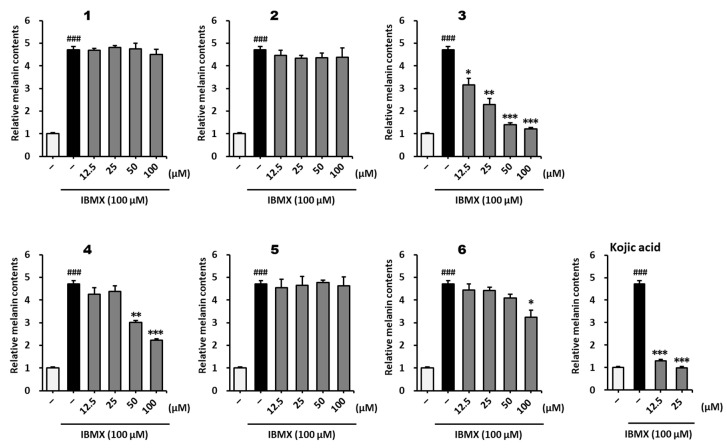
Effect of methoxyflavones **1**–**6** on melanogenesis in B16F10 cells. B16F10 cells were seeded in a 60 mm dish at a density of 5 × 10^5^ cells and incubated for 24 h. The cells were then incubated with IBMX and the indicated concentrations of methoxyflavones **1**–**6** for 72 h. Melanin content was evaluated using 1 N sodium hydroxide. Kojic acid was used as a positive control. The results are presented as the mean ± SEM (*n* =3). ^###^ *p* < 0.001 compared to the untreated group. * *p* < 0.05, ** *p* < 0.01, and *** *p* < 0.001 compared to the IBMX-treated group.

To investigate whether the inhibitory effects of the compounds on cellular tyrosinase influenced melanogenesis, melanin content was measured. As shown in Figure 5, methoxyflavones **3**, **4**, and **6** decreased the melanin content in IBMX-stimulated B16F10 cells. The IBMX-stimulated group showed a 4.72 ± 0.15-fold (*p* < 0.001) increase in melanin content compared to that in the vehicle group. Melanin content decreased in the positive control group treated with kojic acid at 12.5 μM (1.29 ± 0.24-fold, *p* < 0.001) and 25 μM (0.98 ± 0.06-fold, *p* < 0.001) compared with that in the IBMX-treated group. Compound **3** significantly decreased the melanin content at 12.5–100 μM (12.5 μM: 3.16 ± 0.28-fold, *p* < 0.05; 25 μM: 2.30 ± 0.25-fold, *p* < 0.01; 50 μM: 1.41 ± 0.08-fold, *p* < 0.001; 100 μM: 1.21 ± 0.06-fold, *p* < 0.001) compared to that in the IBMX-stimulated group. In addition, Compound **4** decreased melanin content at 50 μM (3.03 ± 0.26-fold, *p* < 0.01) and 100 μM (2.23 ± 0.16-fold, *p* < 0.001) compared to that in the IBMX-stimulated group. Compound **6** weakly inhibited melanin synthesis at 100 μM (3.24 ± 0.31-fold, *p* < 0.001) compared to that in the IBMX-stimulated group. These results indicate that 7,4′-dimethylapigenin (**3**) and 3,5,7-trimethoxyflavone (**4**) significantly inhibited IBMX-stimulated melanin overproduction. Therefore, methoxyflavones derived from *K. parviflora* rhizomes can be said to be effective in reducing melanogenesis.

### 2.4. SAR Analysis

**Figure 6:**
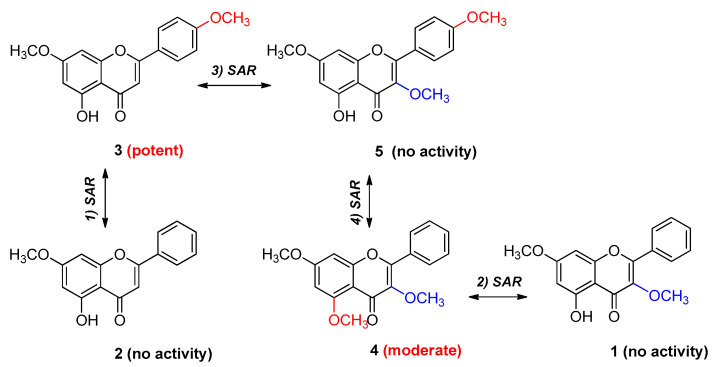
Key structural elements (positive element—red; negative element—blue) influencing anti-melanogenic activity and structure–activity relationships (SARs) among the isolated methoxyflavones.

A better understanding of SARs can lead to the comprehension of the structural characteristics of compounds and the discovery of more potent therapeutic agents to treat and prevent some diseases. SARs have been used to investigate the effects of structural features of molecules on their biological activities; thus, they are considered to be a key tool for drug discovery [44,45,46]. While analyzing the results of anti-melanogenic activity tests, we found interesting SARs among the six methoxyflavones (Figure 6). First, the substitution of the methoxy group at C-4′ in the methoxyflavones enhanced the activity; Compound **3** exhibited the strongest activity, whereas Compound **2** lost its activity without the methoxy group, indicating that the methoxy group at C-4′ is key to anti-melanogenic activity. Second, the substitution of the methoxy group at C-5 in methoxyflavones is a key structural element involved in the activity, based on the moderate activity of Compound **4** and the loss of activity of Compound **1** on substituting a hydroxy group at C-5. Third, the substitution of the methoxy group at C-3 in the methoxyflavones decreased the activity, based on the strongest activity of Compound **3** and the loss of activity in Compound **5** upon substituting a methoxy group at C-3. Lastly, according to the results for Compound **4** and Compound **5**, the methoxy group at C-5 in methoxyflavones had a greater positive effect on the activity than that of the methoxy group at C-4′. The roles of the methoxy groups in the biological activities of flavonoid derivatives are well-known [47,48,49]. Therefore, the anti-melanogenic activity of methoxyflavones depends not only on the number of methoxy groups but also on their position.

## 3. Materials and Methods

### 3.1. Plant Material

*K. parviflora* rhizomes were purchased at Warorot Market in January 2020 from Chiang Mai City, Northern Thailand. One of the authors (K. H. Kim) authenticated the materials, and the voucher specimen (SKKU-BG 1908) was stored in the herbarium at the School of Pharmacy, Sungkyunkwan University, Suwon, Korea.

### 3.2. Extraction and Separation of Methoxyflavones

The dried rhizomes of *K. parviflora* (132 g) were squashed and macerated separately with MeOH and partitioned with various solvents (*n*-hexane, CH_2_Cl_2_, EtOAc, and *n*-BuOH, 700 mL) for 24 h three times at ambient temperature. After that, each organic solvent was evaporated under reduced pressure using a rotary evaporator to obtain four fractions. Four fractions with increasing polarity were obtained: hexane (1.0 g), CH_2_Cl_2_ (3.2 g), EtOAc (0.4 g), and *n*-BuOH-soluble fractions (0.5 g). LC-MS analysis of each fraction indicated that the hexane fraction contained high-quality flavonoids; hence, it was selected for further isolation. LC-MS analysis was conducted using an Agilent 1200 Series HPLC system (Agilent Technologies, Santa Clara, CA, USA) equipped with a diode array detector, 6130 Series ESI mass spectrometer, and an analytical Kinetex C18 100 Å column (100 × 2.1 mm, 5 μm; flow rate: 0.3 mL/min; Phenomenex, Torrance, CA, USA). Thin-layer chromatography was carried out on precoated silica gel F_254_ plates and RP-C_18_ F_254s_ plates (Merck, Darmstadt, Germany), and the plates were visualized under UV light (254 and 365 nm) by heating after spraying with anisaldehyde–sulfuric acid reagent. A portion of the hexane fraction (1.0 g) was chromatographed on a silica gel column with two gradient solvent systems—*n*-hexane/EtOAc (10:1, 3:1, 1:1) and CH_2_Cl_2_/MeOH (10:1, 1:1)—yielding six fractions (Fr.1–Fr.6). Fr.1 (31.1 mg) was subjected to semi-preparative reverse-phase HPLC with 94% MeOH/H_2_O at a flow rate of 2 mL/min, yielding Compound **1** (1.8 mg) (Figure 1). Fr.2 (91.5 mg) was subjected to Sephadex LH-20 column chromatography with an isocratic solvent system comprising CH_2_Cl_2_/MeOH (2:8), yielding five subfractions (Sfr.2.1–Sfr.2.5). Sfr.2.2 (29.2 mg) was further subjected to semi-preparative reverse-phase HPLC with 78% MeOH/H_2_O at a flow rate of 2 mL/min, yielding Compound **2** (4.9 mg). Similarly, Fr.5 (112.7 mg) was also performed on a Sephadex LH-20 column eluting with the same solvent system with Fr.2, yielding two subfractions (Sfr.5.1 and Sfr.5.2). Sfr.5.2 (28.2 mg) was further purified using semi-preparative reverse-phase HPLC with an isocratic solvent (83% MeOH/H_2_O) at a flow rate of 2 mL/min, yielding Compounds **3** (2.2 mg), **4** (2.5 mg), and **5** (3.4 mg). Finally, Fr.6 (271.8 mg) was also fractionated using Sephadex LH-20 column chromatography with an isocratic solvent system comprising CH_2_Cl_2_/MeOH (2:8), yielding four subfractions (Sfr.6.1–Sfr.6.4). Sfr.6.4 (46.3 mg) was efficiently purified using semi-preparative reverse-phase HPLC and eluted with 78% MeOH/H_2_O at a flow rate of 2 mL/min, yielding Compound **6** (3.0 mg).

### 3.3. Cell Culture 

Mouse melanoma B16F10 cells (Korean Cell Line Bank, Seoul, Republic of Korea) were cultured in DMEM medium (Corning, Manassas, VA, USA), supplemented with 10% (*v*/*v*) fetal bovine serum and 1% penicillin/streptomycin, in a humidified atmosphere containing 5% CO_2_ at 37 °C. 

### 3.4. Cell Viability

B16F10 cells were plated in 96-well plates at a density of 5 × 10^3^ cells/well and were grown for 24 h. The following day, cells were treated with each compound (12.5, 25, 50, and 100 μM). After incubation for 24 h, EZ-Cytox solution was added to the culture medium and incubated for 2 h at 37 °C. The absorbance was measured at 450 nm using a microplate reader (SPARK 10M; Tecan, Männedorf, Switzerland).

### 3.5. Measurement of Cellular Tyrosinase Activity

Tyrosinase activity was evaluated using a previous method [50]. B16F10 cells were plated in a 60 mm dish at a density of 5 × 10^5^ cells/dish and grown for 24 h. The following day, the cells were treated with each compound (12.5, 25, 50, and 100 μM) and 100 mM IBMX (Sigma-Aldrich, St. Louis, MO, USA). After incubating for 72 h, the cells were collected and centrifuged. The supernatant was mixed with l-DOPA and incubated at 37 °C for 30 min. The absorbance was measured at 475 nm using a microplate reader (SPARK 10M).

### 3.6. Measurement of Cellular Melanin Content

The melanin content was evaluated using a previous method [51]. B16F10 cells were plated in a 60 mm dish at a density of 5 × 10^5^ cells/dish and grown for 24 h. The following day, cells were treated with each compound (12.5, 25, 50, and 100 μM) and 100 mM IBMX. After incubating for 72 h, the cells were collected and centrifuged. The pellet was collected and lysed with 1 N NaOH containing 10% DMSO at 90 °C for 30 min. The absorbance was measured at 475 nm using a microplate reader (SPARK 10M).

### 3.7. Statistical Analysis

All experiments were conducted in triplicate and are shown as the mean ± SEM. The differences were calculated using one-way analysis of variance, followed by Tukey’s test with GraphPad Prism version 8.0.1 (GraphPad Software Inc., La Jolla, CA, USA). Statistical significance was set at *p* < 0.05. 

## 4. Conclusions

In summary, six methoxyflavones were isolated from the hexane fraction of the MeOH extract of *K. parviflora* rhizomes and characterized using LC-MS analysis. The compounds were identified as 3,7-dimethoxy-5-hydroxyflavone (**1**), 5-hydroxy-7-methoxyflavone (**2**), 7,4′-dimethylapigenin (**3**), 3,5,7-trimethoxyflavone (**4**), 3,7,4′-trimethylkaempferol (**5**), and 5-hydroxy-3,7,3′,4′-tetramethoxyflavone (**6**) using 1D NMR spectroscopic methods, MS data, and LC-MS analysis. In the anti-melanogenic activity assays, Compounds **3** and **4** significantly inhibited tyrosinase hyperactivity and melanin overproduction induced by IBMX. Notably, SAR analysis showed that the methoxy group at C-5 in methoxyflavones is key to their anti-melanogenic activity and that the previously unappreciated methoxy group plays a critical role in the anti-melanogenic activity of flavonoid derivatives. This study provides experimental evidence that *K. parviflora* rhizomes are rich in methoxyflavones and can be a valuable natural resource for anti-melanogenic compounds.

## Data Availability

Date is contained within the article and supplementary material.

## Supplementary Materials

The following are available online at https://www.mdpi.com/article/10.3390/plants12051183/s1: Figure S1: ^1^H-NMR spectrum of Compound **1** (in CDCl_3_); Figure S2: UV chromatogram of LC/MS and UV and MS data for Compound **1**; Figure S3: ^1^H-NMR spectrum of Compound **2** (in CDCl_3_); Figure S4: UV chromatogram of LC/MS and UV and MS data for Compound **2**; Figure S5: ^1^H-NMR spectrum of Compound **3** (in CDCl_3_); Figure S6: UV chromatogram of LC/MS and UV and MS data for Compound **3**; Figure S7: ^1^H-NMR spectrum of Compound **4** (in CDCl_3_); Figure S8: UV chromatogram of LC/MS and UV and MS data for Compound **4**; Figure S9: ^1^H-NMR spectrum of Compound **5** (in CDCl_3_); Figure S10: UV chromatogram of LC/MS and UV and MS data for Compound **5**; Figure S11: ^1^H-NMR spectrum of Compound **6** (in CDCl_3_); Figure S12: UV chromatogram of LC/MS and UV and MS data for Compound **6**.
