# Supplementary material for: Methoxyflavones from Black Ginger (Kaempferia parviflora Wall. ex Baker) and their Inhibitory Effect on Melanogenesis in B16F10 Mouse Melanoma Cells
Source: Plants (Basel). 2023 Mar 5;12(5):1183. doi: 10.3390/plants12051183 (PMC10005586; doi:10.3390/plants12051183)
Supplement: Supplementary file 1 [file plants-12-01183-s001.zip › plants-2210060-supplementary.pdf]

Supplementary Materials

Methoxyflavones from Black Ginger (*Kaempferia parviflora* Wall. ex Baker) and their Inhibitory Effect on Melanogenesis in B16F10 Mouse Melanoma Cells

Chen Huo [1,†], Sullim Lee [2,†], Min Jeong Yoo [1], Bum Soo Lee [1], Yoon Seo Jang [1], Ho Kyong Kim [3], Seulah Lee [1,4], Han Yong Bae [5,*] and Ki Hyun Kim [1,*]

[1] School of Pharmacy, Sungkyunkwan University, Suwon 16419, Republic of Korea
[2] Department of Life Science, College of Bio-Nano Technology, Gachon University, Seongnam 13120, Republic of Korea
[3] STL company, Yongin 17086, Republic of Korea
[4] Department of Oriental Medicine Biotechnology, College of Life Sciences, Kyung Hee University, Yongin 17104, Republic of Korea
[5] Department of Chemistry, Sungkyunkwan University, Suwon 16419, Republic of Korea
* Correspondence: hybae@skku.edu (H.Y.B.); khkim83@skku.edu (K.H.K.); Tel.: +82-31-290-7700 (K.H.K.)
† These authors contributed equally to this study.

Figure S1 : ^{1}H-NMR spectrum of compound **1** (in $CDCl_3$)
Figure S2 : UV chromatogram of LC/MS and UV and MS data for compound **1**
Figure S3 : ^{1}H-NMR spectrum of compound **2** (in $CDCl_3$)
Figure S4 : UV chromatogram of LC/MS and UV and MS data for compound **2**
Figure S5 : ^{1}H-NMR spectrum of compound **3** (in $CDCl_3$)
Figure S6 : UV chromatogram of LC/MS and UV and MS data for compound **3**
Figure S7 : ^{1}H-NMR spectrum of compound **4** (in $CDCl_3$)
Figure S8 : UV chromatogram of LC/MS and UV and MS data for compound **4**
Figure S9 : ^{1}H-NMR spectrum of compound **5** (in $CDCl_3$)
Figure S10 : UV chromatogram of LC/MS and UV and MS data for compound **5**
Figure S11 : ^{1}H-NMR spectrum of compound **6** (in $CDCl_3$)
Figure S12 : UV chromatogram of LC/MS and UV and MS data for compound **6**

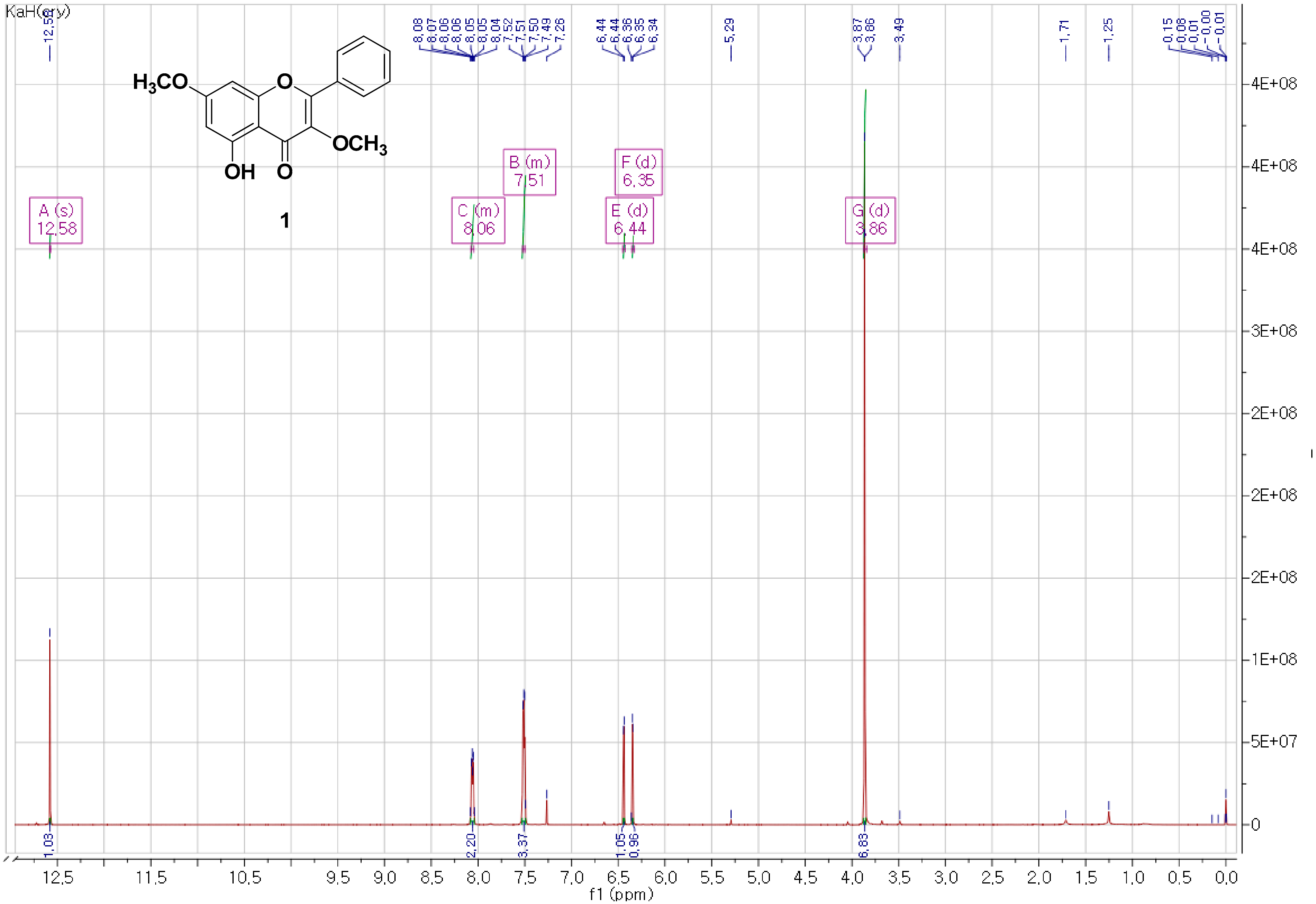

Figure S1 : ^{1}H-NMR spectrum of compound **1** (in $CDCl_3$)

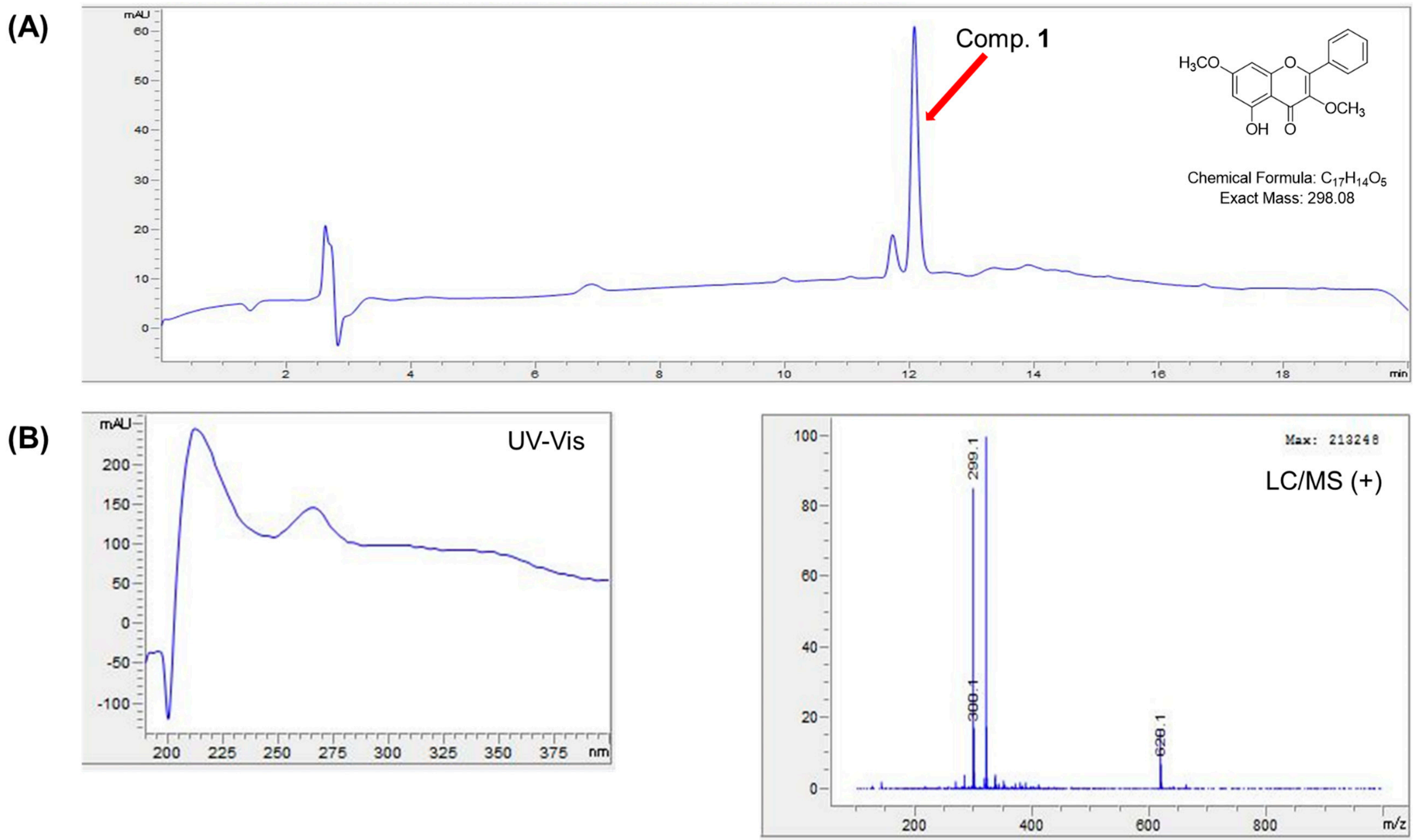

Figure S2 : (A) UV chromatogram of LC/MS (detection wavelength: 254 nm) and (B) UV and MS data for compound **1**

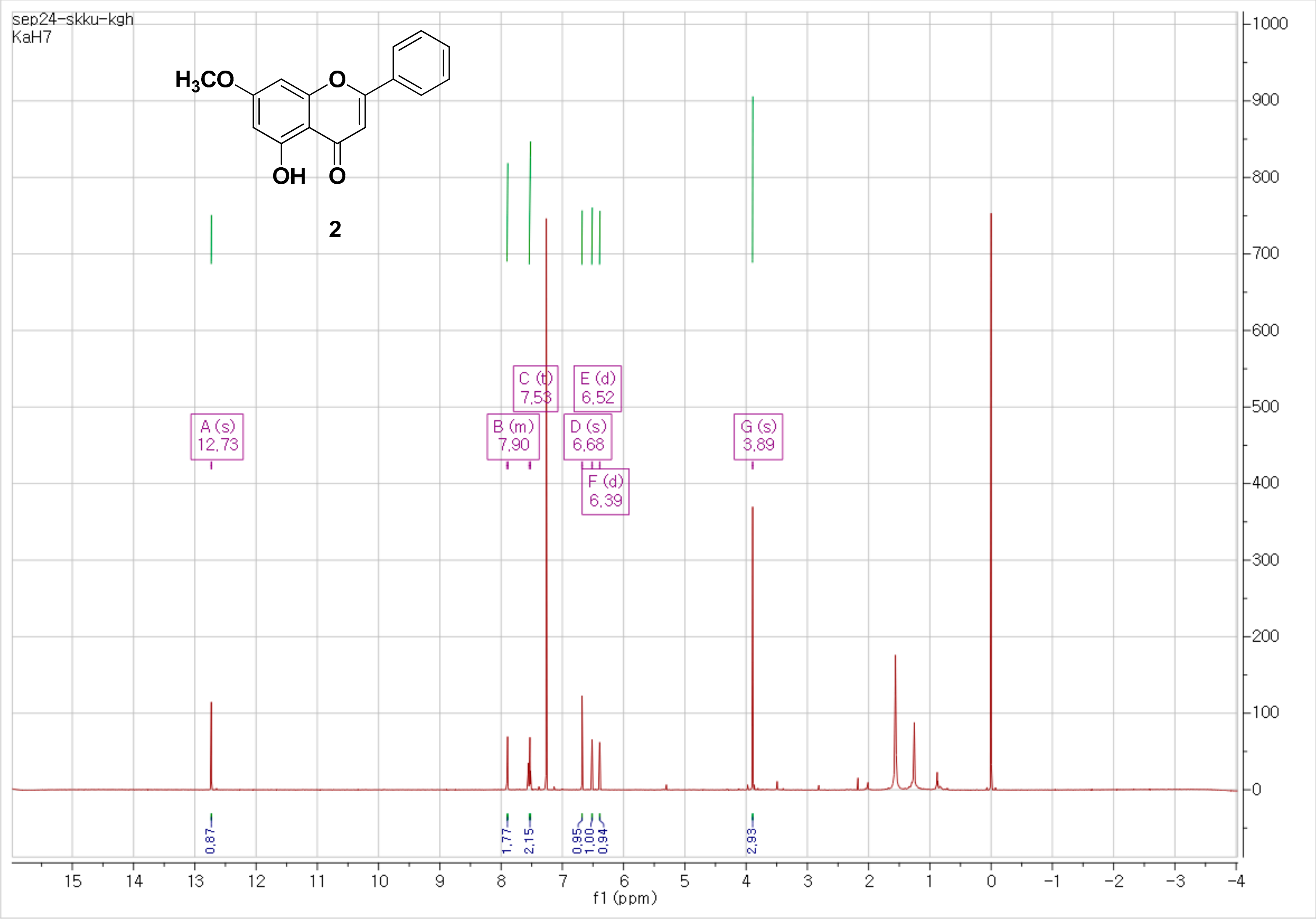

Figure S3 : ^{1}H-NMR spectrum of compound **2** (in $CDCl_3$)

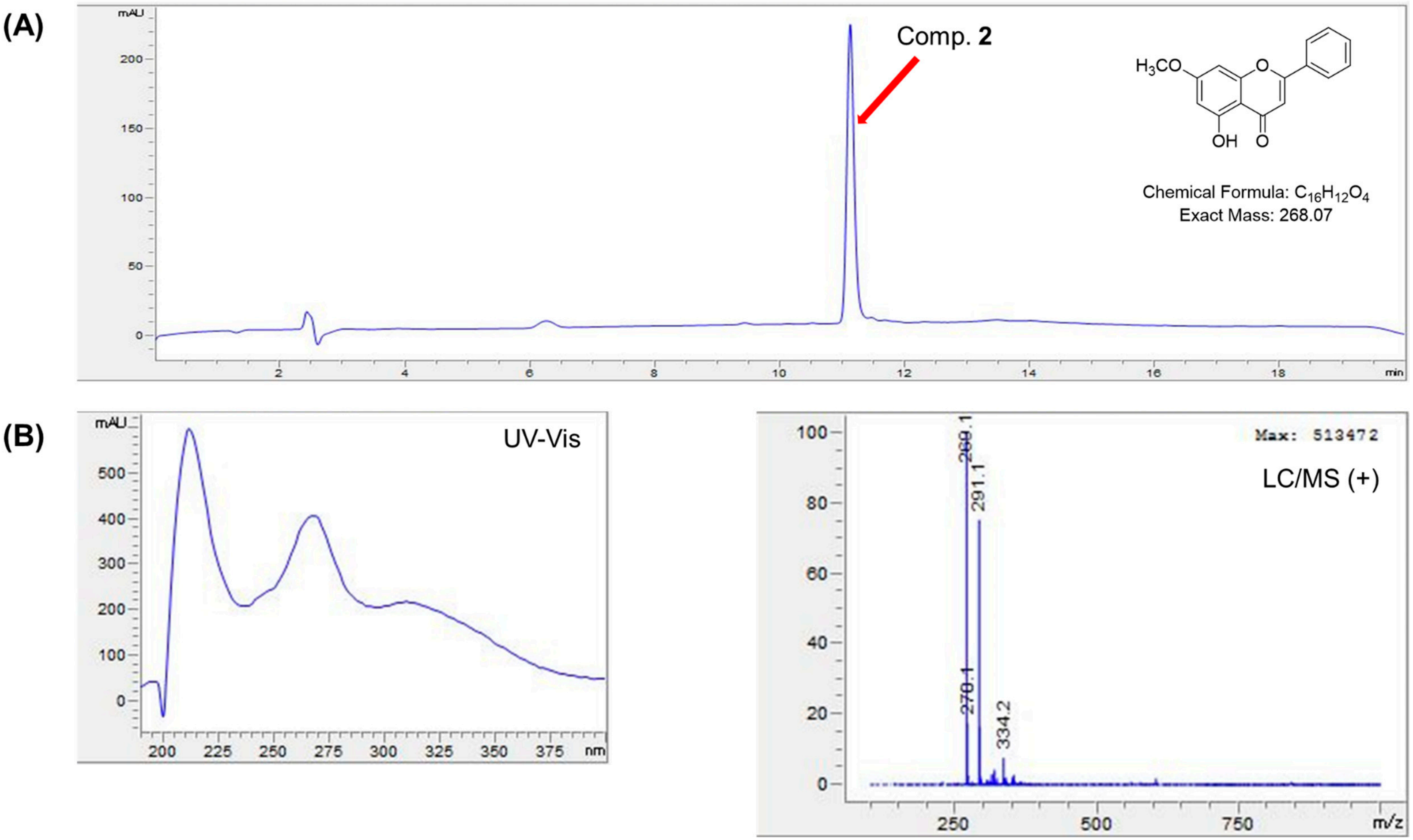

Figure S4 : (A) UV chromatogram of LC/MS (detection wavelength: 254 nm) and (B) UV and MS data for compound **2**

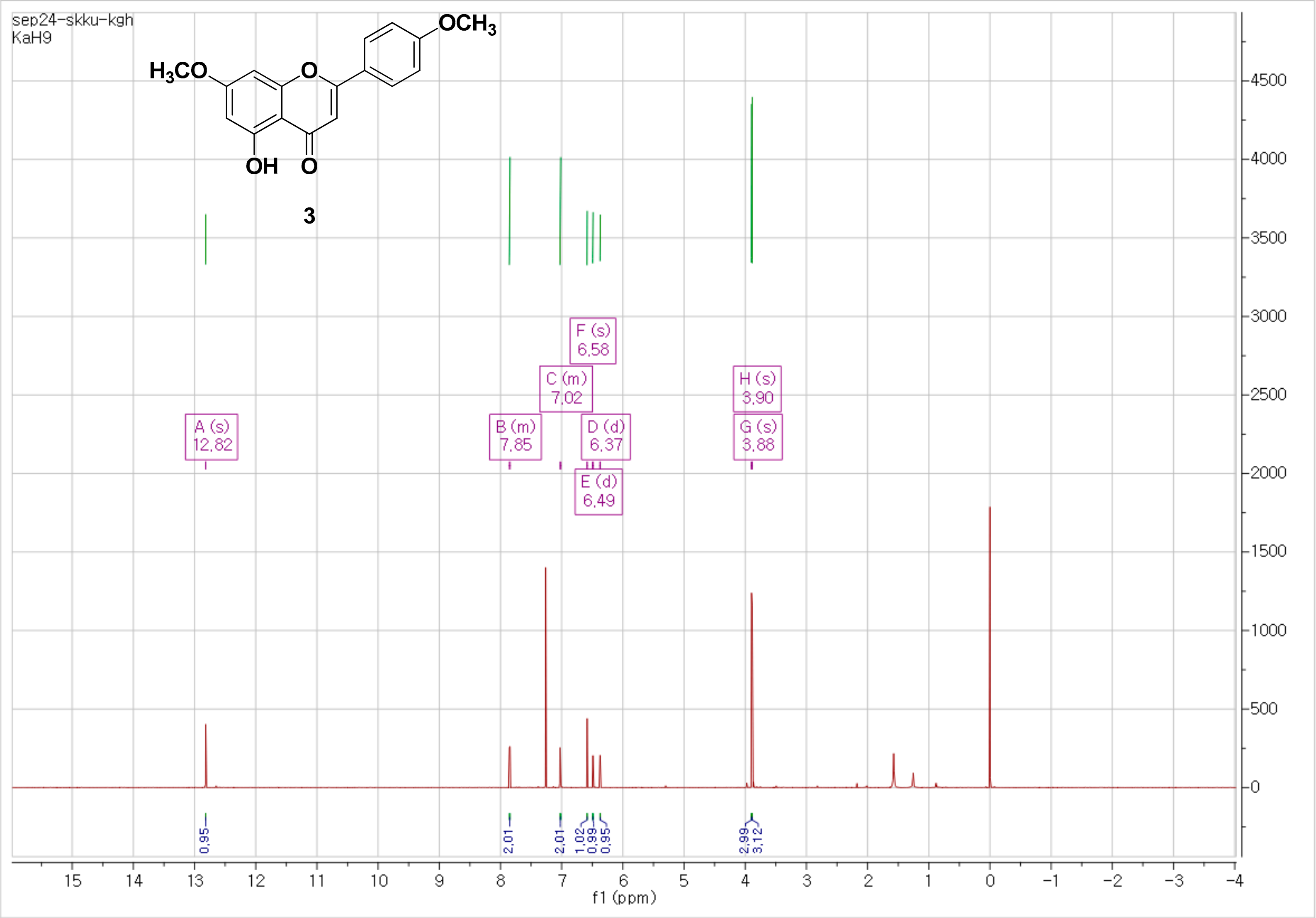

Figure S5 : ^{1}H-NMR spectrum of compound **3** (in CDCl3)

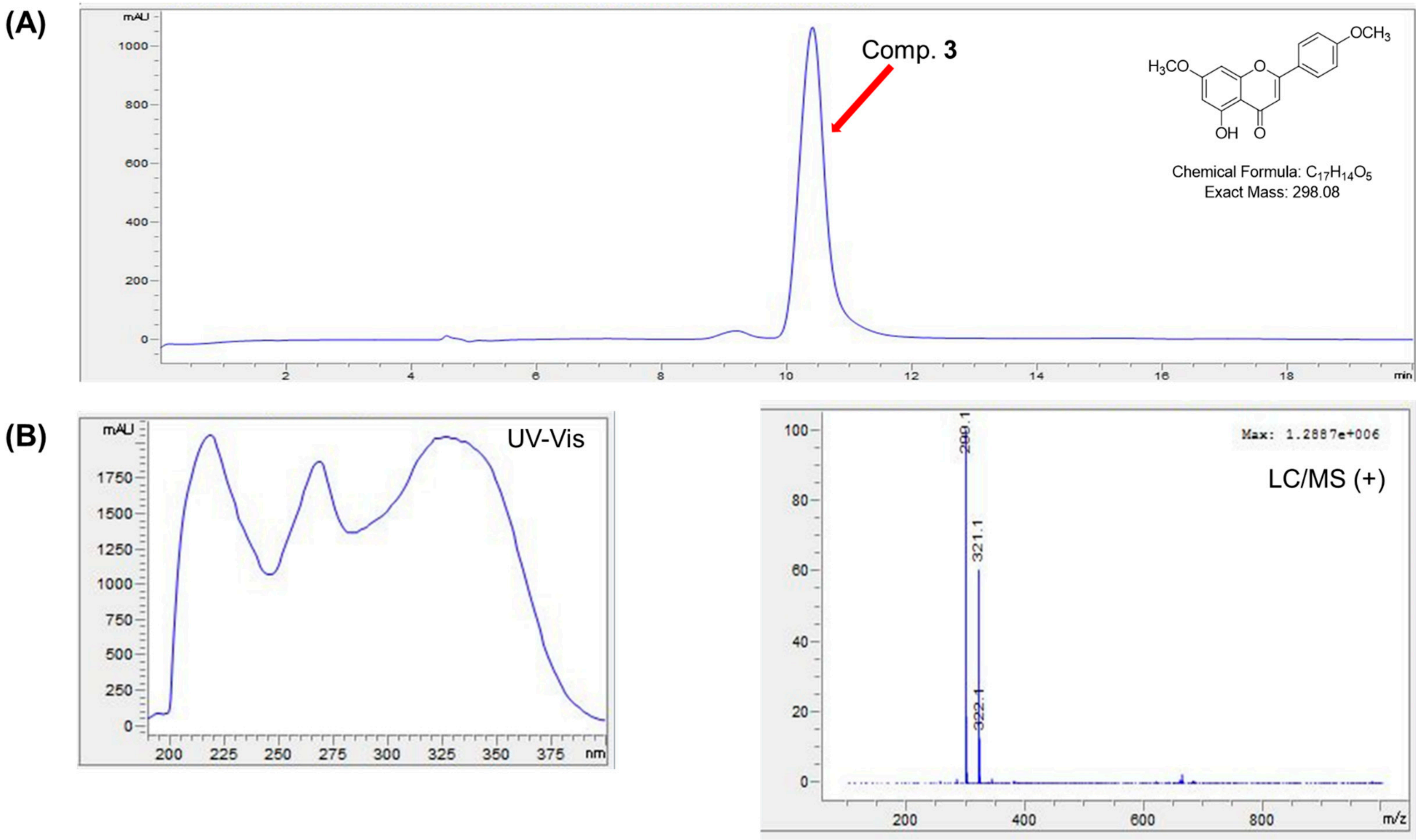

Figure S6 : (A) UV chromatogram of LC/MS (detection wavelength: 254 nm) and (B) UV and MS data for compound **3**

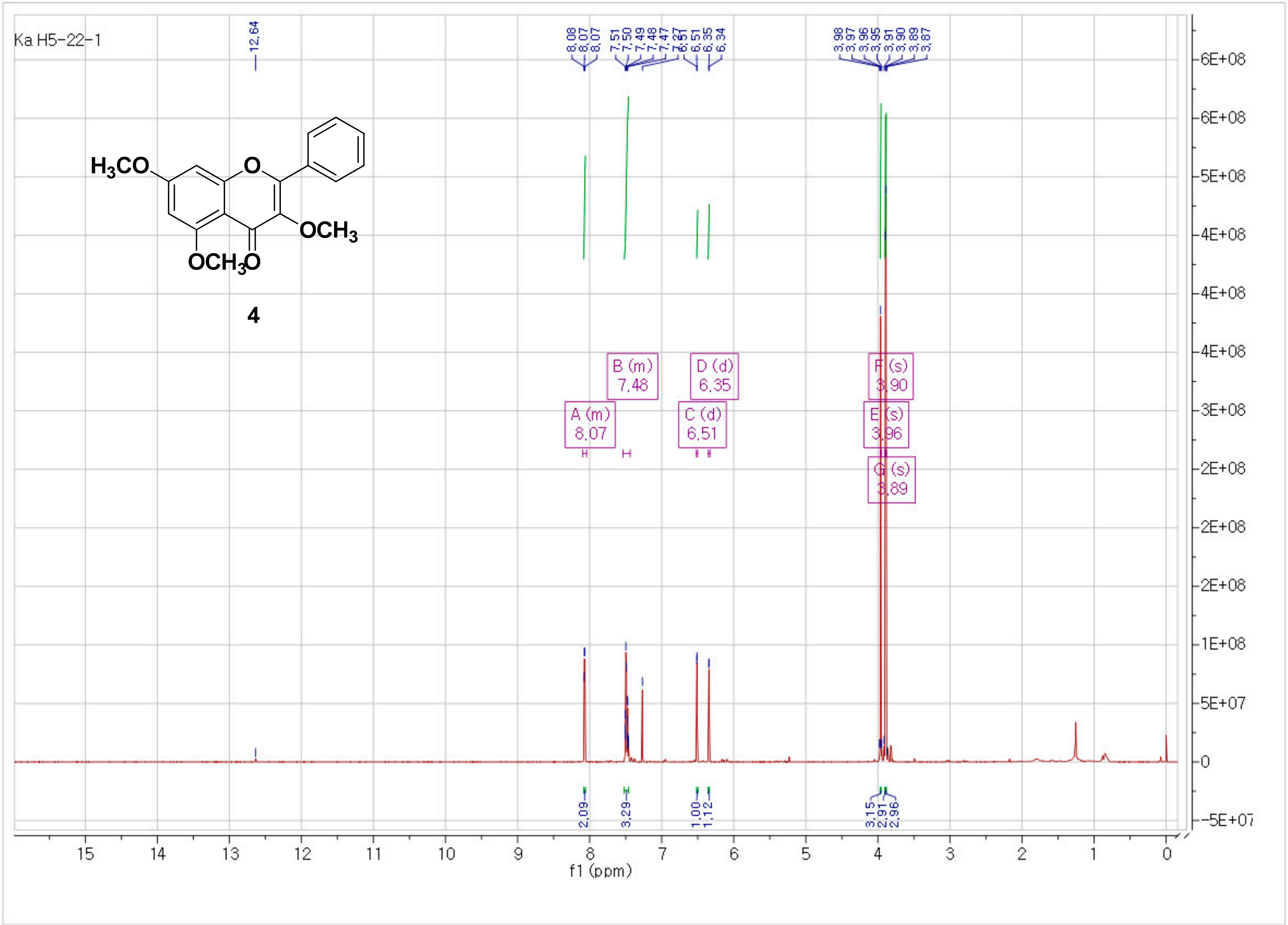

Figure S7 : ^{1}H-NMR spectrum of compound **4** (in $CDCl_3$)

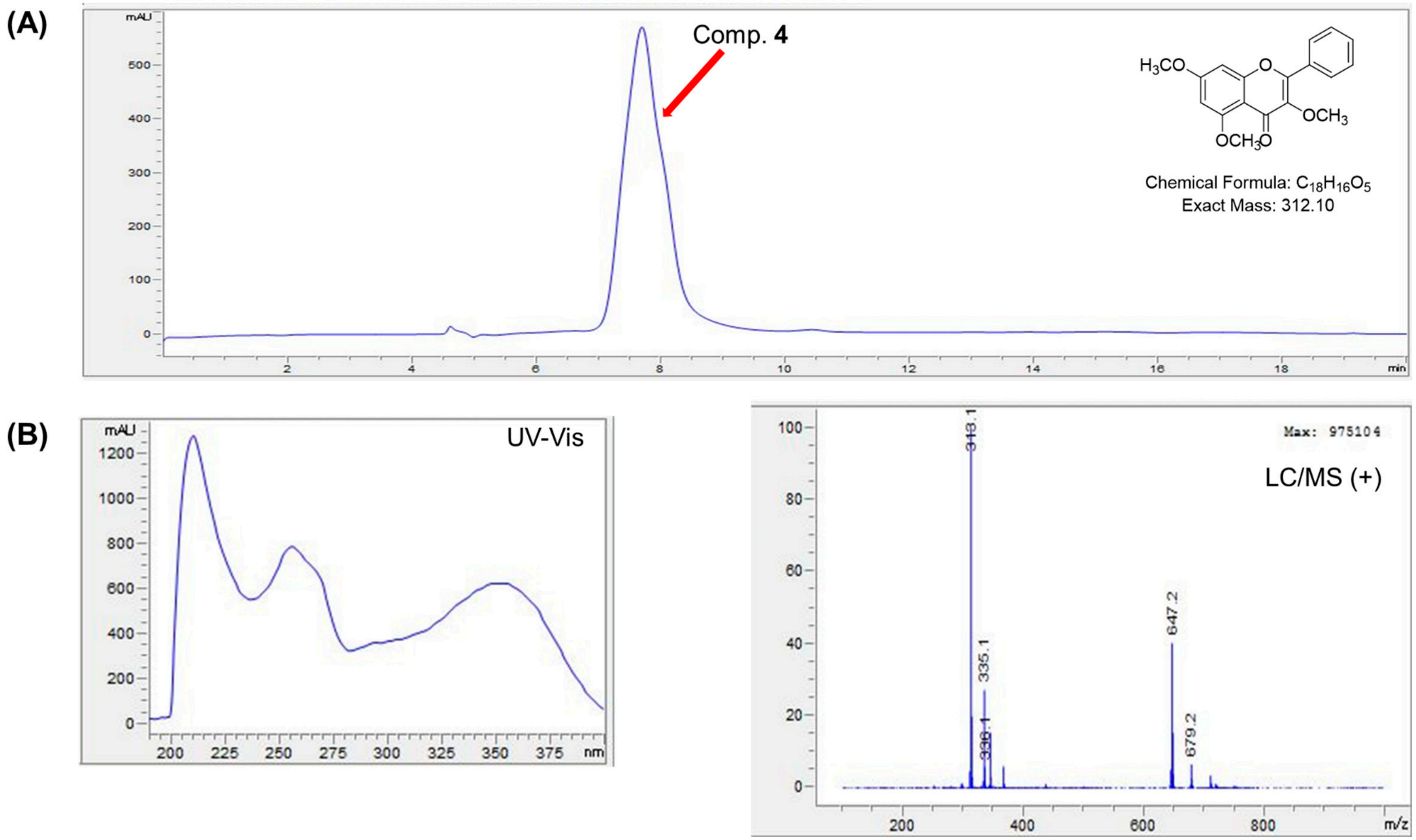

Figure S8 : (A) UV chromatogram of LC/MS (detection wavelength: 254 nm) and (B) UV and MS data for compound **4**

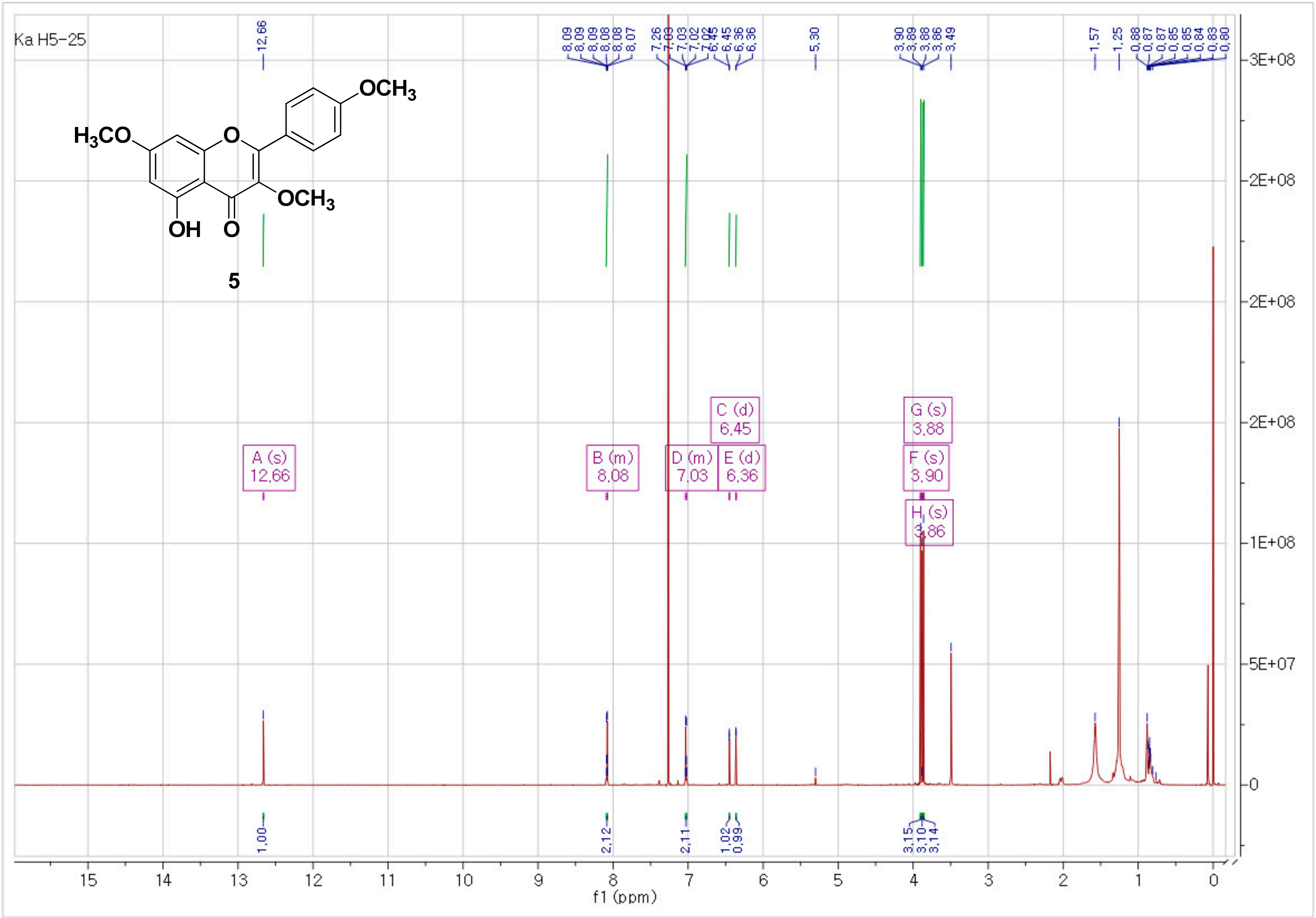

Figure S9 : ^{1}H-NMR spectrum of compound **5** (in $CDCl_3$)

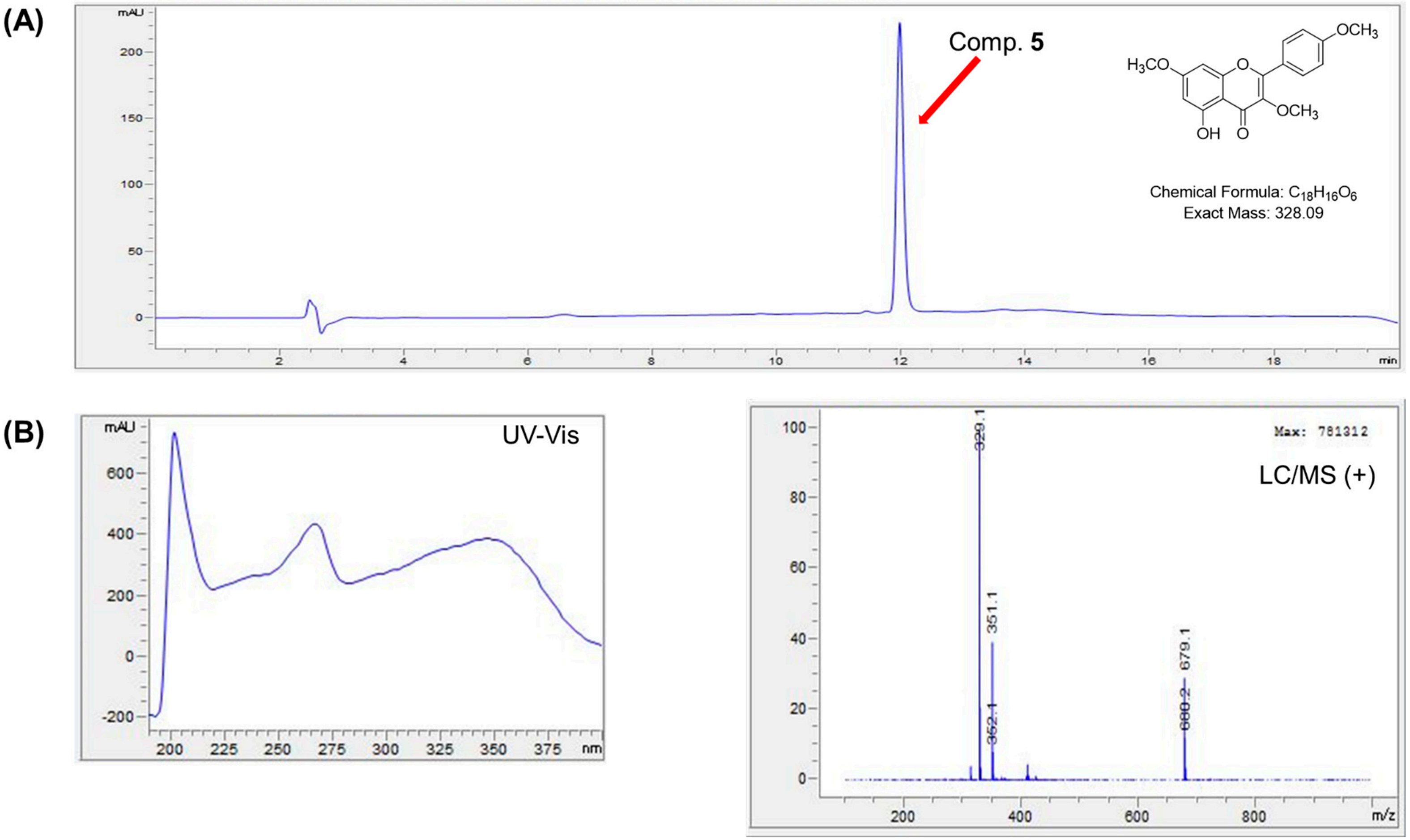

Figure S10 : (A) UV chromatogram of LC/MS (detection wavelength: 254 nm) and (B) UV and MS data for compound **5**

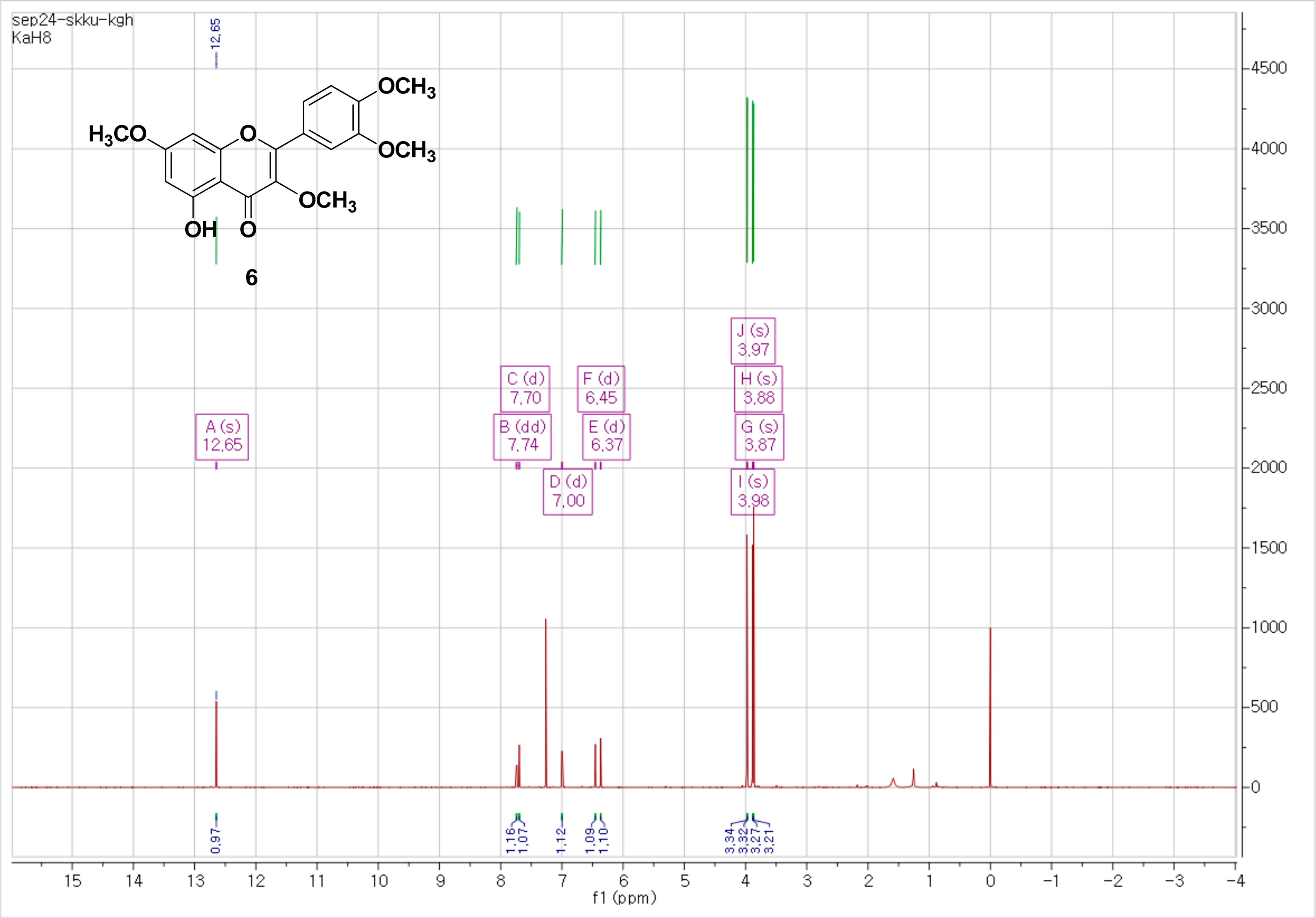

Figure S11 : ^{1}H-NMR spectrum of compound **6** (in $CDCl_3$)

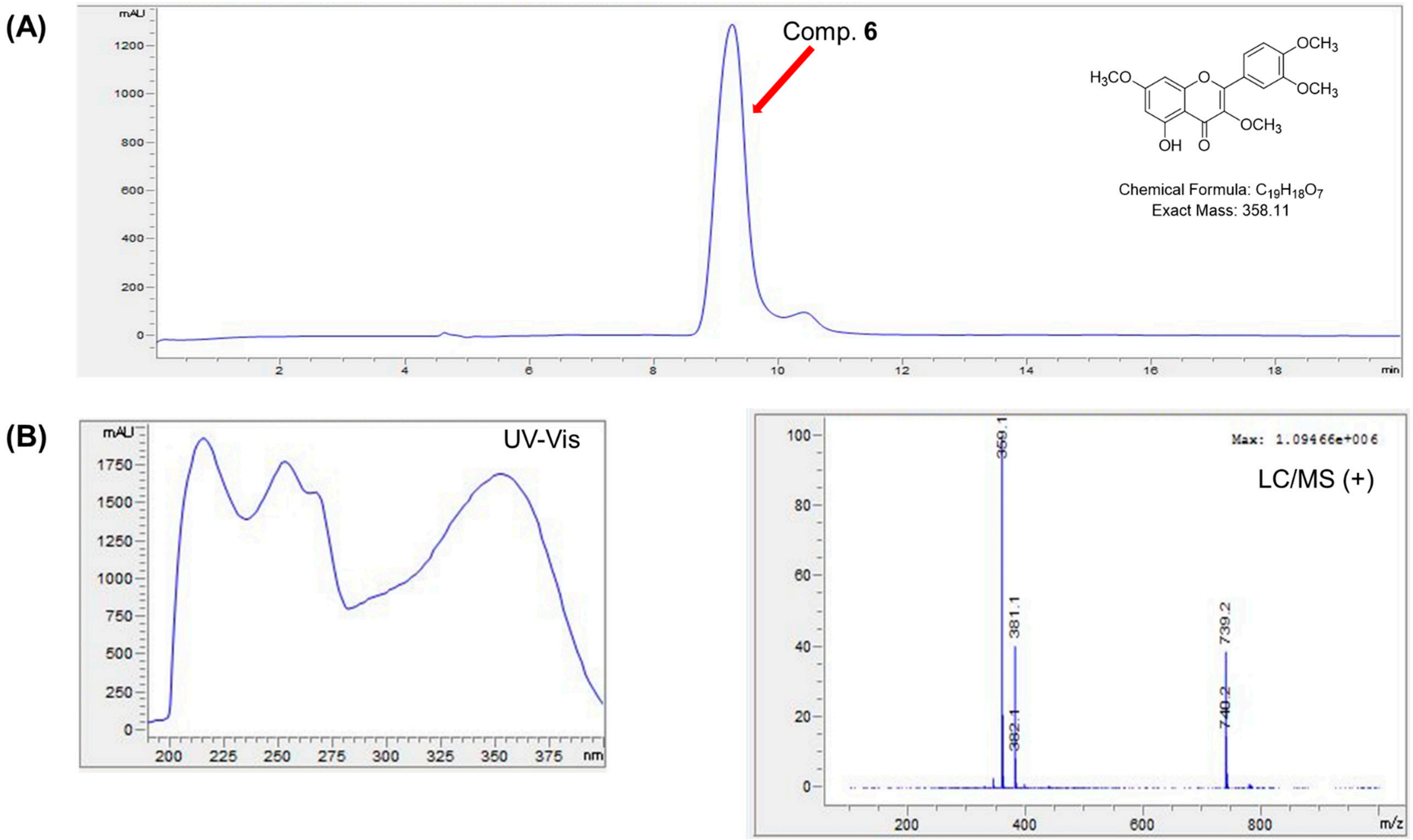

Figure S12 : (A) UV chromatogram of LC/MS (detection wavelength: 254 nm) and (B) UV and MS data for compound **6**
